# The association between parental risks and childhood development: findings from a community-based survey in East China

**DOI:** 10.1186/s12889-023-15702-y

**Published:** 2023-05-12

**Authors:** Shuangshuang Zheng, Jianing Fang, Guannan Bai, Xinyu He, Mengdi Hua, Bingquan Zhu, Weijun Chen, Wenhong Dong, Lei Wang, Xiaona Huang, Huishan Wang, Jie Shao

**Affiliations:** 1 Department of Child Health Care, Children’s Hospital, Zhejiang University School of Medicine, National Clinical Research Center for Child Health, 57 Zhu-Gan Road, Gongshu District, 310003 Hangzhou, China; 2 Section of Child Health and Development, the United Nations Children’s Fund, UNICEF Office for China, Beijing, China; 3 Department of Children Health, National Center for Maternal and Children Health, Chinese Center for Disease Control and Prevention, Beijing, China

**Keywords:** Nurturing care, Parental risk, Early Childhood Development, Developmental Delay, Neurodevelopment

## Abstract

**Background:**

Nurturing care is necessary for optimal early childhood development. This study aimed to investigate the prevalence of parental risks in rural East China and assess their impacts on early development in children younger than three years old.

**Methods:**

This community-based cross-sectional survey was conducted among 3852 caregiver-child pairs in Zhejiang Province from December 2019 to January 2020. Children aged 0 to 3 years were recruited from China’s Early Childhood Development Program (ECD). Local child health care providers conducted face-to-face interviews with the primary caregivers. Demographic information of the participants was collected by questionnaire. Each child was screened for parental risk through the Parental Risk Checklist designed by the ECD program. The Ages and Stages Questionnaire (ASQ) was used to identify children with potential developmental delays. Multinomial logistic regression model and linear trend test were applied to assess the association between parental risks and suspected developmental delays.

**Results:**

Among the 3852 children included in the analyses, 46.70% had at least one parental risk and 9.01% presented suspected developmental delays in any domain of ASQ. Parental risk was statistically associated with the overall suspected developmental delay in young children (Relative Risk Ratio (RRR): 1.36; 95% confidence interval (CI): 1.08, 1.72; P = 0.010) after adjusting potential confounders. Compared with children with no parental risk, children exposed to 3 or more parental risks had 2.59, 5.76, 3.95, and 2.84 times higher risk of the suspected developmental delay in overall ASQ, communication, problem-solving, and personal-social domain, respectively (P values < 0.05). The linear trend tests found that the more parental risks, the higher possibility of developmental delay (P values < 0.05).

**Conclusions:**

Parental risks are prevalent among children under three years in rural East China, which may increase the risk of developmental delays in children. Meanwhile, parental risk screening can be used to recognize poor nurturing care in primary health care settings. Targeted interventions are warranted to improve nurturing care for optimal early childhood development.

**Supplementary Information:**

The online version contains supplementary material available at 10.1186/s12889-023-15702-y.

## Background

Early childhood, especially the first 1,000 days, is the most significant developmental period during which the foundations are laid for optimal health, growth, and neurodevelopment throughout the lifespan [1]. Meanwhile, early childhood is also a vulnerable period when inappropriate nurturing environments can have short- and long-term effects on achieving children’s potential. Therefore, a safe, responsive, and loving nurturing environment with adequate nutrition and stimulation from caregivers is critical for early development. However, it is estimated that approximately 250 million children (43%) under five years of age in low and middle-income countries failed to reach their full developmental potential due to poverty, malnutrition, and lack of appropriate stimulation or access to healthcare [2]. And in poverty-stricken areas of China, up to 39.7% of children younger than three years were at risk of developmental delays [3].

Extensive researches show that early childhood development interventions can promote health equity [4], improve educational achievement, reduce crime and violence [5, 6], and improve health and economic productivity in adulthood [7, 8]. Also, investment in early childhood development is more cost-effective than at any other time [9] and substantially benefits individuals and nations. Therefore, providing children with high-quality nurturing care has become a global priority to ensure children survive and thrive, including good health, adequate nutrition, responsive caregiving, opportunities for early learning, and security and safety [10]. The early detection of young children at risk of poor nurturing care is vital to providing individual interventions.

China, the world’s second-largest economy, announced the eradication of extreme poverty in 2020. However, China is still grappling with economic and social inequality. The urban-rural gap in child health status still exists. The lowest provincial mortality rate of under-five in China was lower than that of developed countries such as Canada, New Zealand, and the USA, while the highest provincial level was higher than that of developing countries like Bangladesh [11]. Risk factors affecting early childhood development have received sustained attention in poverty areas, especially in middle western China [3, 12, 13]. The Early Childhood Development (ECD) Program of China has been implemented in poverty-stricken areas since 2013. Community-based integrated nurturing care practices and services were developed to promote early childhood development by using a range of channels, including parental consultation, home visits, and care group activities [3, 12]. In 2019, the National Health Commission of China scaled up the ECD Program to 26 counties of 10 provinces, including some economically developed provinces such as Zhejiang province which is located on the eastern coast of China. Despite the high economic level in Zhejiang province, knowledge about the state of nurturing care and early childhood development was limited. Therefore, it is necessary to conduct a baseline survey of the ECD Program particularly to address the data gap on the status of nurturing care and early childhood development. This study aimed to investigate the prevalence of parental risks and assess the association between parental risks and children’s developmental outcomes. The results of our research will also provide baseline data to evaluate the effectiveness of the ECD Program in rural Eastern China.

## Methods

### Study population

A community-based cross-sectional survey was conducted among children under three years old and their caregivers in four counties (i.e., Ninghai, Yiwu, Wenling, and Jingning) in Zhejiang Province where the ECD Program was launched. According to the Zhejiang Provincial Bureau of Statistics data, per capita gross domestic product (GDP) of Zhejiang Province was 100,620 RMB in 2020. The per capita GDP of Ninghai is the same as that of the provincial level, while the per capita GDP of the other three counties (Yiwu, Wenling, and Jingning) is about 70–80% of the provincial level.

According to the design of the ECD Program, about half of the townships (33/69) from the four counties were randomly selected as project sites. We randomly selected 14 ECD project townships and another 14 non-ECD project townships in the four counties, based on the per capita GDP and the numbers of children under three years old matched each other. Finally, 28 townships participated in the baseline survey of the ECD Program.

Based on the child health care system of China, primary child health care services are provided free for each child under six years old, including newborn visits, newborn screening, hearing screening, vaccination, routine child health examinations at 1, 3, 6, 8, 12, 18, 24, 30 months and once a year later, and follow-up management of high-risk infants and nutritional diseases. During the routine child health examination, physical examination, growth and development monitoring and promotion, anemia screening, vision, hearing, and oral healthcare are provided for each child at community health centers according to the child health care standards issued by the National Health Commission of the People’s Republic of China [14–16].

In this study, Children aged 1–35 months who attended primary child health care at the community health centers in the selected 28 townships were eligible for recruitment from December 2019 to January 2020. Inclusion criteria included (1) child aged 1–35 months; (2) local permanent residents; (3) attending primary health care visits as scheduled. Those who refused to participate in or participate in other child intervention programs were excluded. Qualified children were recruited by local child health care providers until the minimum sample size required was reached.

This study was conducted according to the Declaration of Helsinki [17], and approved by the Ethics Committee of Children’s Hospital, Zhejiang University School of Medicine (2020-IRB-084). Written informed consent was obtained from the parents or caregivers of the all children.

### Data collection

Face-to-face interviews with the mothers or primary caregivers were conducted by qualified local child health care providers, who were trained and supervised by the project personnel from the Children’s Hospital, Zhejiang University School of Medicine. We used a self-developed questionnaire to collect demographic information about the participants and their families, including the age and sex of the child, gestational age at birth and birth weight, age and educational level of parents, household income, and family structure. Family structure was categorized as a nuclear family (father, mother, and children), three-generation family (grandparents, father, mother, and children), extended family (grandparents, parents, children, and other relatives), and single-parent family (father or mother and children).

### Parental risk screening

The Parental Risk Checklist was developed for parental risk screening by the ECD Program in 2019. Items of the Checklist cover five domains of nurturing care (health, nutrition, responsive caregiving, opportunities for early learning, and security and safety) according to three different age groups (0–5 months, 6–12 months, and 1–3 years of age). There are ten items in the Checklist for each age group (Supplementary Tables 1–3). Malnutrition in the Checklist includes stunting, wasting, underweight, and anemia. If the caregiver gave a positive answer in any item, it indicated a risk of nurturing care. We also considered the number of positive items.

### Developmental delay screening

Ages and Stages Questionnaire Chinese version (ASQ-C) [18], a parent-completed standardized screening tool, was used to identify children with potential developmental delay in five domains: communication, gross motor, fine motor, problem-solving, and personal-social domain. The result of each domain [18] is evaluated as above the cutoff point (the score above the mean (X) − 1 standard deviation (SD)), close to the cutoff point (the score above X − 2SD and below X − 1SD), and below the cutoff point (the score below X-2SD). A score below the cutoff point in any domain indicates suspected developmental delays in a child, while a score close to the cutoff point in any domain indicates that a child is on marginal status of suspected development delay.

### Sample size

The sample size was determined based on the prevalence of developmental delay, using the formula: ${\rm{N = }}\frac{{{\rm{\mu }}_{{\rm{\alpha /2}}}^{\rm{2}}{\rm{*\pi (1 - \pi )}}}}{{{{\rm{\delta }}^{\rm{2}}}}}$. N will be 2827 with an alpha of 5% (µ_α/2_ = 1.96), an error (δ) of 0.01, and the prevalence of suspected developmental delay (π) of 8% according to the results of our pilot survey. Assuming that the missing rate is 20%, 3393 subjects are needed. Finally, 130 subjects are required for each survey site.

### Statistical analysis

The distributions of demographic characteristics, parental risks, and suspected developmental delays in overall ASQ and five domains among different ages were described by frequency and percentage for the categorical variables, and mean and standard deviation for the continuous variables. A multinomial logistic regression model was applied to assess the association between parental risks and suspected developmental delays adjusted by potential confounders such as age and gender of the child, age and educational level of parents, household income, and family structure. In addition, a linear trend of the number of parental risks and suspected developmental delays was tested. All analyses were performed using Statistical Analysis System software version 9.2 (SAS Institute Inc, Cary, North Carolina). The statistical significance was indicated by a P value < 0.05.

## Results

### General characteristics of study participants

Among 3869 caregiver-child pairs surveyed, 3852 pairs who completed the parental risk screening and ASQ were finally included for analyses. Table 1 presents the general characteristics of the study participants. The mean age of children was 15.02 (SD: 9.42) months and 51.71% were males. Over half of the parents were educated below high school. Nuclear family or three-generation family accounted for around 45%, respectively. About 67% of families had a gross household income lower than 150,000 RMB per year. In this population, 75% of the mothers took care of their children as primary caregivers.

**Table 1:** General characteristics of children, caregivers, and families

Characteristics	N (%)
Mean age of children, months*	15.02 ± 9.42
Age group of children	
0–5 months	600(15.57)
6–12 months	1442(37.44)
1–3 years	1810(46.99)
Gender of children	
Male	1992 (51.71)
Female	1860 (48.29)
Mean age of fathers, years*	33.30 ± 5.55
Mean age of mothers, years*	31.16 ± 5.10
Educational level of fathers	
High school or below	2228 (57.84)
College or above	1624 (42.16)
Educational level of mothers	
High school or below	2020 (52.44)
College or above	1832 (47.56)
Primary caregivers	
Mother	2874 (74.61)
Other	978 (25.39)
Educational level of Caregivers	
High school or below	2651 (68.82)
College or above	1201 (31.18)
Family structure	
Nuclear family	1750 (45.43)
Three generation family	1739 (45.15)
Extended or single-parent	363 (9.42)
Family income per year, RMB	
0-50000	373 (9.68)
50,000–90,000	973 (25.26)
100,000–140,000	1248 (32.40)
≥ 150,000	1193 (30.97)
unknown	65 (1.69)

*Values presented as mean ± standard deviation

### Parental risks

Table 2 shows the parental risks among different age groups of children. Overall, children with at least one parental risk accounted for 46.70% of the total children surveyed in this study, and this percentage was 43.83% for children aged 0–5 months, 54.16% for children aged 6–12 months, and 41.71% for children aged 1–3 years. Among all participants, 30.84% of children had one risk item, 11.06% had two risk items, and 4.80% had three or more risk items. The top three parental risk factors in children aged 0–5 months were no breastfeeding, toys less than three, and premature and/or low birth weight. Among children aged 6–12 months, the top three risks were caregivers rarely talking or reading stories to the child, malnutrition, and caregivers rarely playing with the child. Caregivers rarely talking or reading stories to the child, no nutritional supplements, and caregivers rarely playing with the child were the significant risks for children aged 1–3 years.

**Table 2:** Parental risks among different age children

Item	0–5 months	6–12 months	1–3 years
Overall risk	263(43.83)	781(54.16)	755(41.71)
Premature and/or low birth weight	42(7.00)	NA*	NA
Hospitalized for more than two weeks during the neonatal period	8(1.33)	NA	NA
No breastfeeding	119(19.83)	NA	NA
No breastfeeding or milk	NA	31(2.15)	NA
No supplementary foods	NA	107(7.42)	NA
Rarely eating meat or eggs	NA	NA	62(3.43)
No nutritional supplements	NA	104(7.21)	306(16.91)
Rarely talking, laughing, or playing with the child	10(1.67)	NA	NA
Leaving the child alone for more than an hour	39(6.50)	70(4.85)	NA
Rarely responding to child’s cries or other sounds in time	10(1.67)	NA	NA
Rarely playing with the child	NA	134(9.29)	93(5.14)
Rarely talking or reading stories to the child	NA	490(33.98)	327(18.07)
Toys less than three	90(15.00)	51(3.54)	39(2.15)
No picture books	NA	NA	84(4.64)
Motherless care	4(0.67)	10(0.69)	NA
Easily access to hot water, pesticides, etc.	NA	NA	9(0.50)
Punishing the child frequently	NA	NA	84(4.64)
Positive in warning sign	8(1.33)	18(1.25)	56(3.09)
Malnutrition	25(4.17)	153(10.61)	79(4.36)

NA means the item is not applicable in this age group

### Developmental status

Table 3 shows the children’s developmental status among different age groups. Nearly 9.01% of children presented suspected developmental delay in any domain of ASQ, while 19.44% of children were on the marginal status of suspected development delay. The corresponding prevalence of children with suspected overall developmental delays was 21.50% for children aged 0–5 months, 10.26% for children aged 6–12 months, and 3.87% for children aged 1–3 years respectively, which decreased with age (P < 0.001). The children with suspected developmental delays in the communication, gross motor, fine motor, problem-solving, and personal-social domain accounted for 1.27%, 3.53%, 2.75%, 1.71%, and 4.49%, respectively.

**Table 3:** Developmental delays in overall ASQ and five domains among different age children

Domain	Whole sample	0–5 months	6–12 months	1–3 years
Overall				
Normal	2756 (71.55)	303 (50.50)	975 (67.61)	1478 (81.66)
Marginal status	749 (19.44)	168 (28.00)	319 (22.12)	262 (14.48)
Suspected Delay	347 (9.01)	129 (21.50)	148 (10.26)	70 (3.87)
Communication				
Normal	3650 (94.76)	489 (81.50)	1409 (97.71)	1752 (96.80)
Marginal status	153 (3.97)	85 (14.17)	26 (1.80)	42 (2.32)
Suspected Delay	49 (1.27)	26 (4.33)	7 (0.49)	16 (0.88)
Gross motor				
Normal	3381 (87.77)	466 (77.67)	1182 (81.97)	1733 (95.75)
Marginal status	335 (8.70)	83 (13.83)	187 (12.97)	65 (3.59)
Suspected Delay	136 (3.53)	51 (8.50)	73 (5.06)	12 (0.66)
Fine motor				
Normal	3465 (89.95)	462 (77.00)	1321 (91.61)	1682 (92.93)
Marginal status	281 (7.29)	95 (15.83)	88 (6.10)	98 (5.41)
Suspected Delay	106 (2.75)	43 (7.17)	33 (2.29)	30 (1.66)
Problem-solving				
Normal	3548 (92.11)	452 (75.33)	1342 (93.07)	1754 (96.91)
Marginal status	238 (6.18)	113 (18.83)	77 (5.34)	48 (2.65)
Suspected Delay	66 (1.71)	35 (5.83)	23 (1.60)	8 (0.44)
Personal-social				
Normal	3241 (84.14)	412 (68.67)	1186 (82.25)	1643 (90.77)
Marginal status	438 (11.37)	115 (19.17)	183 (12.69)	140 (7.73)
Suspected Delay	173 (4.49)	73 (12.17)	73 (5.06)	27 (1.49)

### Parental risk and childhood development

Table 4 presents the associations between parental risks and developmental delays. Among children aged 0–3 years old, the parental risk was significantly associated with overall suspected developmental delay indicated (RRR: 1.36; 95% CI: 1.08, 1.72; P = 0.010) adjusted by potential confounders. As for children aged 6–12 months, the parental risk was associated with suspected developmental delay in the problem-solving domain (RRR: 3.04; 95% CI: 1.10, 8.46; P = 0.033). While for children aged 1–3 years, the parental risk was positively associated with suspected developmental delay in overall ASQ (RRR: 2.51; 95% CI: 1.50, 4.21; P = 0.001), communication (RRR: 4.61; 95% CI: 1.27, 16.72; P = 0.020), fine motor (RRR: 2.20; 95% CI: 1.01, 4.77; P = 0.046), and personal-social domain (RRR: 3.14, 95%CI: 1.33, 7.41; P = 0.009). There was no statistically significant association between parental risks and suspected developmental delays among children aged 0–5 months. The association between parental risks and suspected developmental delays in overall ASQ (RRR: 1.51, 95%CI: 1.11, 2.07; P = 0.010) and the personal-social domain (RRR: 1.56, 95% CI: 1.02, 2.38; P = 0.043) were also found in boys, but not in girls (Supplementary Table 4).

**Table 4:** Association between parental risks and developmental delays by age group

Age group	Domain	Marginal status			Suspected Developmental delays	
		RRR (95%CI)	P value		RRR (95%CI)	P value
0–3 years	Overall	1.16 (0.98,1.37)	0.087		1.36 (1.08,1.72)	0.010
	Communication	0.86 (0.62,1.20)	0.377		1.70 (0.94,3.09)	0.081
	Gross motor	1.12 (0.89,1.42)	0.334		1.24 (0.87,1.78)	0.235
	Fine motor	1.05 (0.82,1.34)	0.722		1.06 (0.72,1.58)	0.764
	Problem-solving	1.07 (0.82,1.41)	0.617		1.11 (0.67,1.83)	0.682
	Personal-social	1.22 (0.99,1.49)	0.062		1.37 (0.99,1.88)	0.055
0–5 months	Overall	0.96 (0.65,1.43)	0.853		0.90 (0.58,1.39)	0.619
	Communication	0.71 (0.43,1.17)	0.179		1.45 (0.62,3.35)	0.391
	Gross motor	1.15 (0.71,1.87)	0.578		0.80 (0.43,1.49)	0.486
	Fine motor	1.05 (0.65,1.69)	0.849		0.99 (0.51,1.92)	0.965
	Problem-solving	1.26 (0.82,1.93)	0.296		0.53 (0.25,1.14)	0.105
	Personal-social	1.05 (0.68,1.64)	0.813		0.65 (0.38,1.13)	0.125
6–12 months	Overall	0.95 (0.73,1.24)	0.708		1.22 (0.84,1.76)	0.301
	Communication	0.79 (0.35,1.79)	0.573		0.59 (0.13,2.73)	0.496
	Gross motor	0.89 (0.64,1.23)	0.480		1.42 (0.85,2.37)	0.186
	Fine motor	1.07 (0.68,1.67)	0.774		0.73 (0.36,1.49)	0.389
	Problem-solving	0.87 (0.54,1.40)	0.560		3.04 (1.10,8.46)	0.033
	Personal-social	0.94 (0.68,1.32)	0.736		1.60 (0.94,2.73)	0.084
1–3 years	Overall	1.38 (1.05,1.81)	0.021		2.51 (1.50,4.21)	0.001
	Communication	1.52 (0.81,2.87)	0.197		4.61 (1.27,16.72)	0.020
	Gross motor	1.55 (0.93,2.57)	0.093		0.90 (0.27,3.02)	0.870
	Fine motor	1.43 (0.94,2.17)	0.097		2.20 (1.01,4.77)	0.046
	Problem-solving	1.41 (0.78,2.54)	0.259		2.93 (0.55,15.62)	0.208
	Personal-social	1.33 (0.93,1.91)	0.117		3.14 (1.33,7.41)	0.009

The above models were adjusted by age and gender of the child, age and educational level of the mother, age and educational level of the father, family structure and income

Table 5 shows the results of a dose-response analysis between the number of parental risks and the risk of developmental delays. Compared to the children with no parental risk, the children exposed to three or more parental risks had 2.59, 5.76, 3.95, and 2.84 times higher risk of suspected developmental delays in overall ASQ, communication, problem-solving, and personal-social domain (P values < 0.05), respectively. The linear trend tests found that the more parental risk factors, the higher possibility of developmental delay (P values < 0.05).

**Table 5:** Association of the numbers of parental risks with developmental delays

Domain	Number of risks	Marginal status			Suspected Developmental delays	
		RRR (95%CI)	P value		RRR (95%CI)	P value
Overall	0	REF	-		REF	-
	1	1.12 (0.93,1.35)	0.224		1.23 (0.94,1.60)	0.128
	2	1.17 (0.90,1.54)	0.241		1.32 (0.91,1.91)	0.142
	≥ 3	1.37 (0.93,2.02)	0.111		2.59 (1.63,4.10)	< 0.001
	P trend		0.052			< 0.001
Communication	0	REF	-		REF	-
	1	0.82 (0.56,1.21)	0.319		1.36 (0.68,2.70)	0.382
	2	0.72 (0.41,1.27)	0.258		1.07 (0.39,2.95)	0.894
	≥ 3	1.55 (0.80,3.01)	0.198		5.76 (2.55,12.98)	< 0.001
	P trend		0.920			0.002
Gross motor	0	REF	-		REF	-
	1	1.11 (0.86,1.44)	0.434		1.11 (0.74,1.66)	0.620
	2	1.05 (0.72,1.54)	0.790		1.46 (0.85,2.50)	0.171
	≥ 3	1.39 (0.83,2.34)	0.212		1.77 (0.83,3.75)	0.140
	P trend		0.268			0.075
Fine motor	0	REF	-		REF	-
	1	0.99 (0.75,1.32)	0.945		1.02 (0.65,1.60)	0.921
	2	1.18 (0.80,1.73)	0.400		1.05 (0.56,1.97)	0.888
	≥ 3	1.11 (0.62,1.98)	0.726		1.38 (0.60,3.13)	0.448
	P trend		0.495			0.566
Problem-solving	0	REF	-		REF	-
	1	1.12 (0.83,1.51)	0.464		0.71 (0.38,1.34)	0.291
	2	0.78 (0.49,1.25)	0.306		1.30 (0.62,2.74)	0.483
	≥ 3	1.57 (0.88,2.81)	0.128		3.95 (1.83,8.54)	0.001
	P trend		0.613			0.011
Personal-social	0	REF	-		REF	-
	1	1.15 (0.91,1.45)	0.238		1.13 (0.78,1.63)	0.523
	2	1.18 (0.85,1.64)	0.323		1.56 (0.98,2.50)	0.064
	≥ 3	1.83 (1.19,2.82)	0.006		2.84 (1.57,5.14)	0.001
	P trend		0.012			0.001

The above regression models were adjusted by age and gender of child, age and educational level of mother, age and educational level of father, family structure and income

## Discussion

With the decline in under-five mortality and child health improvement, global strategies for child health have shifted from survival to thriving. The evidence of cost-efficiency and long-term benefits of appropriate nurturing care on child development identified strong economic values for investment in early childhood, especially in the first three years.

Zhejiang province is one of the most economically developed provinces in China, where urban-rural gaps in economy and child health are smaller than that in other regions. However, our results found that about 47% of children younger than three years were at risk of poor nurturing care, and 16% had two or more parental risks. For children younger than six months, the top three parental risks were no breastfeeding, fewer toys, and premature and/or low birth weight. While for children aged 6–35 months, the main parental risks were inadequate nutrition (malnutrition and no nutritional supplements) and a lack of early childhood stimulation (fewer toys, rarely talking, reading stories, or playing with the child). Poor nurturing care was associated with suspected developmental delays, especially in boys and children aged 1–3 years. We also identified the dose-response relationship between parental risks and increased suspected developmental delays. Our study supports that parental risk screening can be used to recognize poor nurturing care in primary healthcare settings. It would provide opportunities for early detection and individual interventions for children at risk of developmental delay.

Regarding parental risks, no breastfeeding and preterm birth were the main risks for children younger than six months. Numerous studies have confirmed that breastfeeding has clear benefits for children, such as reducing mortality and morbidity from infectious diseases, promoting cognitive development [19], reducing behavioral disorders [20], and increasing educational attainment and income in adulthood [21]. Preterm birth rates have increased in many countries [22]. Although neonatal intensive care practices have markedly improved the survival of premature infants, preterm birth remains the leading cause of death in children younger than five years [23] and increases the risk of language, cognitive, sensory, motor, hearing, and vision deficits as well as behavioral problems [24]. Early developmental interventions provided post-hospital discharge for preterm infants improved the cognitive and motor outcomes during infancy and the cognitive benefits could persist into preschool age [25]. In addition, our research data found that 11% of children aged 6–12 months suffered from malnutrition, and 17% of children aged 1–3 years did not receive nutritional supplements. Malnutrition and micronutrient deficiencies are the major risks undermining children’s survival, growth, and development in developing countries [10]. Iron and micronutrient supplementation has been proven to improve children’s nutritional status, promote psychomotor development, and improve academic performance [26, 27]. Our findings suggest that we should promote breastfeeding and enhance follow-up management by providing nutritional and developmental interventions for preterm, malnutrition, and micronutrient deficiencies in infants.

Early environments with little responsive stimulation, such as fewer toys or books, and less parent-child interaction, emerged as significant parental risks in our study population. The effect became more pronounced with increasing age. Providing stimulating environments or early learning opportunities for young children plays a crucial role in early brain development. The American Academy of Pediatrics (AAP) recommended that pediatricians should guide families in selecting appropriate toys for young children [28] and promote the developmental benefits of play [29]. A prospective, longitudinal cohort study in Canada showed that informal play opportunities, picture books, or being cared for in childcare centers could protect toddlers from late talking [30]. Findings from the China ECD Program also presented that a lack of books and toys [31] and insufficient learning activities [3] significantly increased the risk of developmental delays among children aged 0–35 months in rural areas. Additionally, a longitudinal birth cohort study revealed that responsive caregiving and learning opportunities could protect young children against the effects of early adversities on adolescent human capital [32].

This study also indicated the impact of parental risks on children’s development, which might have accumulation effects and differences in gender and age. Early childhood, especially 1–3 years is a time of rapid language and social-emotional development that may be vulnerable to parental risks. Poor nurturing environments, such as maternal depression, lower parenting self-efficacy, non-play-based interaction, and nonattendance in playgroups, possibly delayed social-emotional development and further led to behavioral problems [33]. Our study also suggested poor nurturing care increased the risk of suspected developmental delays in communication and personal-social domains in children aged 1–3 years, and in the problem-solving domain in children aged 6–12 months. Furthermore, the more parental risks that a child experienced, the higher risk of developmental delay might occur. However, no significant associations were found in children younger than six months possibly due to the limited sample size and the cumulative effect of parental risks over time. Studies have found that boys were more vulnerable to developmental harm from poor nurturing care [30, 34]. Boys were more likely to be diagnosed with developmental difficulties, specifically with attention-deficit/ hyperactivity disorder, autism spectrum disorder, cerebral palsy, learning disability, intellectual disability stuttering [34], and language delay [30]. Early testosterone exposure and parental socialization might work together to generate gender differences in the human brain and behaviors [35]. Whether there are age-specific or gender-specific parental risks on children’s development and the mechanisms warrant further studies.

Over the past few decades, many early childhood development interventions delivered by face-to-face health consultation, home visits, or caregiver-child group activities have been developed to promote positive parenting behaviors and the optimal development of young children [36]. In Pakistan, integrated responsive stimulation and nutrition interventions positively affected children’s cognitive, language, motor, and social-emotional development at 12 or 24 months of age [37]. Implementing the ECD intervention in rural China can effectively improve the nurturing care environment, including enhancing caregivers’ mental health, optimizing child feeding and early stimulation behaviors, reducing violent discipline [38], and further reducing the risk of developmental delays [12]. Thus, the early detection of children at risk by applying the Parental Risk Checklist would enable those children timely access to targeted interventions and services to improve the nurturing care for optimal early childhood development.

To our best knowledge, this is one of the few community-based studies to investigate nurturing care and early childhood development in such a large sample. Our findings help to confirm the significance and applicability of parental risk screening in economically well-developed regions of China, particularly for primary health care. However, there are still some limitations to be aware of. First, due to the current cross-sectional study design, it is impossible to draw causal conclusions between parental risks and suspected developmental delays. Longitudinal studies are needed to confirm the causality further. Second, the research was conducted in one province, which might not necessarily represent the whole of China. Third, the Parental Risk Checklist still needs to be standardized for further use. In addition, children with suspected developmental delays in this study are identified by ASQ, a standardized screening tool. Diagnostic evaluations are required to clarify the development of children.

## Conclusions

Parental risks for children under age three are prevalent in rural East China. Poor nurturing care may increase the risk of suspected developmental delays and may have cumulative effects. Parental risk screening can be used to recognize poor nurturing care in primary health care settings. We call for the attention of policymakers and health professionals that there is a great need to implement interventions to improve the nurturing care environment and eventually benefit optimal early childhood development.

## Electronic supplementary material

Below is the link to the electronic supplementary material.

Supplementary Material 1

## Data Availability

Data supporting the findings of this study are not publicly available due to protect the privacy and confidentiality of participants in this study but are available upon reasonable request to the corresponding author.
