# Supplementary material for: The association between parental risks and childhood development: findings from a community-based survey in East China
Source: BMC Public Health. 2023 May 12;23:878. doi: 10.1186/s12889-023-15702-y (PMC10176942; doi:10.1186/s12889-023-15702-y)
Supplement: Supplementary file 1 — Supplementary Material 1 [file 12889_2023_15702_MOESM1_ESM.docx]

**Supplementary Table 1 Parental Risk Checklist（＜6 months）**

Name： Gender：Male□ Female□ Birthday： Age：

| **No.** | **Risk Factors** | **Positive（√）** | |
| --- | --- | --- | --- |
|  |  | **1**  **month** | **3**  **month** |
| 1 | Premature(＜37 weeks) and/or low birth weight (＜2500g) |  |  |
| 2 | Hospitalized for more than two weeks during the neonatal period |  |  |
| 3 | No breastfeeding |  |  |
| 4 | Rarely talking, laughing, or playing with the child |  |  |
| 5 | Leaving the child alone for more than an hour |  |  |
| 6 | Rarely responding to child's cries or other sounds in time |  |  |
| 7 | Toys less than three（including homemade toys） |  |  |
| 8 | Motherless care |  |  |
| 9 | Positive in warning sign for children mental and behavioral development checklist screening |  |  |
| 10 | Malnutrition（anemia and stunting etc.） |  |  |

**Supplementary Table 2 Parental Risk Checklist（6-<12 months）**

Name： Gender：Male□ Female□ Birthday： Age：

| **No.** | **Risk Factors** | **Positive（√）** | | |
| --- | --- | --- | --- | --- |
|  |  | **6 month** | **8 month** | **12 month** |
| 1 | No breastfeeding or milk |  |  |  |
| 2 | No supplementary foods (Such as iron fortified rice flour or meat puree) |  |  |  |
| 3 | No nutritional supplements（Vitamin D, Vitamin AD, Iron, calcium or zinc etc.） |  |  |  |
| 4 | Rarely playing with the child |  |  |  |
| 5 | Leaving the child alone for more than an hour |  |  |  |
| 6 | Rarely talking or reading stories to the child |  |  |  |
| 7 | Toys less than three（including homemade toys） |  |  |  |
| 8 | Motherless care |  |  |  |
| 9 | Positive in warning sign for children mental and behavioral development checklist screening |  |  |  |
| 10 | Malnutrition（anemia and stunting etc.） |  |  |  |

Supplementary Table 3 Parental Risk Checklist（1-3 years）

Name： Gender：Male□ Female□ Birthday： Age：

| **No.** | **Risk Factors** | **Positive（√）** | | |
| --- | --- | --- | --- | --- |
|  |  | **1.5**  **years** | **2**  **years** | **2.5**  **years** |
| 1 | Rarely eating meat or eggs |  |  |  |
| 2 | No nutritional supplements （Vitamin D, Vitamin AD, Iron, calcium or zinc etc.） |  |  |  |
| 3 | Rarely playing with the child |  |  |  |
| 4 | Rarely talking or reading stories to the child |  |  |  |
| 5 | Toys less than three（including homemade toys） |  |  |  |
| 6 | No picture books |  |  |  |
| 7 | Easily access to hot water, pesticides, etc. |  |  |  |
| 8 | Punishing the child frequently |  |  |  |
| 9 | Positive in warning sign for children mental and behavioral development checklist screening |  |  |  |
| 10 | Malnutrition（anemia and stunting etc.） |  |  |  |

| Supplementary Table 4 Association between parental risks and developmental delays by gender | | | | | | |
| --- | --- | --- | --- | --- | --- | --- |
| Gender | Domain | Marginal status | |  | Suspected Developmental delays | |
|  |  | RRR (95%CI) | P value |  | RRR (95%CI) | P value |
| Males | Overall | 1.18 (0.94,1.48) | 0.154 |  | 1.51 (1.11,2.07) | 0.010 |
|  | Communication | 0.81 (0.51,1.28) | 0.363 |  | 1.74 (0.83,3.62) | 0.140 |
|  | Gross motor | 1.25 (0.91,1.71) | 0.172 |  | 1.16 (0.70,1.90) | 0.571 |
|  | Fine motor | 1.26 (0.89,1.78) | 0.201 |  | 1.30 (0.77,2.18) | 0.324 |
|  | Problem-solving | 1.22 (0.84,1.76) | 0.293 |  | 1.59 (0.82,3.11) | 0.173 |
|  | Personal-social | 1.17 (0.89,1.54) | 0.274 |  | 1.56 (1.02,2.38) | 0.043 |
| Females | Overall | 1.13 (0.88,1.45) | 0.329 |  | 1.20 (0.84,1.73) | 0.322 |
|  | Communication | 0.92 (0.57,1.49) | 0.733 |  | 1.71 (0.61,4.79) | 0.309 |
|  | Gross motor | 1.00 (0.70,1.42) | 0.988 |  | 1.36 (0.81,2.28) | 0.252 |
|  | Fine motor | 0.87 (0.60,1.25) | 0.439 |  | 0.80 (0.43,1.51) | 0.491 |
|  | Problem-solving | 0.92 (0.61,1.39) | 0.693 |  | 0.72 (0.32,1.60) | 0.414 |
|  | Personal-social | 1.30 (0.95,1.77) | 0.098 |  | 1.17 (0.72,1.92) | 0.524 |
